# Evolutionary dynamics of the severe acute respiratory syndrome coronavirus 2 genomes

**DOI:** 10.1515/mr-2021-0035

**Published:** 2022-03-01

**Authors:** Zhaohui Qian, Pei Li, Xiaolu Tang, Jian Lu

**Affiliations:** NHC Key Laboratory of Systems Biology of Pathogens, Institute of Pathogen Biology, Chinese Academy of Medical Sciences and Peking Union Medical College, Beijing, 100871, China; State Key Laboratory of Protein and Plant Gene Research, Center for Bioinformatics, School of Life Sciences, Peking University, Beijing, 100176, China

**Keywords:** molecular evolution, population genetics, severe acute respiratory syndrome coronavirus 2, variant, virus

## Abstract

The coronavirus disease 2019 (COVID-19) pandemic has caused immense losses in human lives and the global economy and posed significant challenges for global public health. As severe acute respiratory syndrome coronavirus 2 (SARS-CoV-2), the causative agent of COVID-19, has evolved, thousands of single nucleotide variants (SNVs) have been identified across the viral genome. The roles of individual SNVs in the zoonotic origin, evolution, and transmission of SARS-CoV-2 have become the focus of many studies. This review summarizes recent comparative genomic analyses of SARS-CoV-2 and related coronaviruses (SC2r-CoVs) found in non-human animals, including delineation of SARS-CoV-2 lineages based on characteristic SNVs. We also discuss the current understanding of receptor-binding domain (RBD) evolution and characteristic mutations in variants of concern (VOCs) of SARS-CoV-2, as well as possible co-evolution between RBD and its receptor, angiotensin-converting enzyme 2 (ACE2). We propose that the interplay between SARS-CoV-2 and host RNA editing mechanisms might have partially resulted in the bias in nucleotide changes during SARS-CoV-2 evolution. Finally, we outline some current challenges, including difficulty in deciphering the complicated relationship between viral pathogenicity and infectivity of different variants, and monitoring transmission of SARS-CoV-2 between humans and animals as the pandemic progresses.

## The structure, composition, replication and transcription of the SARS-CoV-2 genome

The coronavirus disease 2019 (COVID-19) pandemic has caused immense losses in human lives and the global economy and posed significant challenges for global public health. As of December 14th, 2021, there were over 270 million confirmed cases and 5.32 million reported deaths [1]. The SARS-CoV-2, a newly identified sarbecovirus in the genus Betacoronavirus (β-CoVs or Beta-CoVs), is the causative agent of COVID-19 pandemic [2], [3], [4], [5].

**Figure 1:**
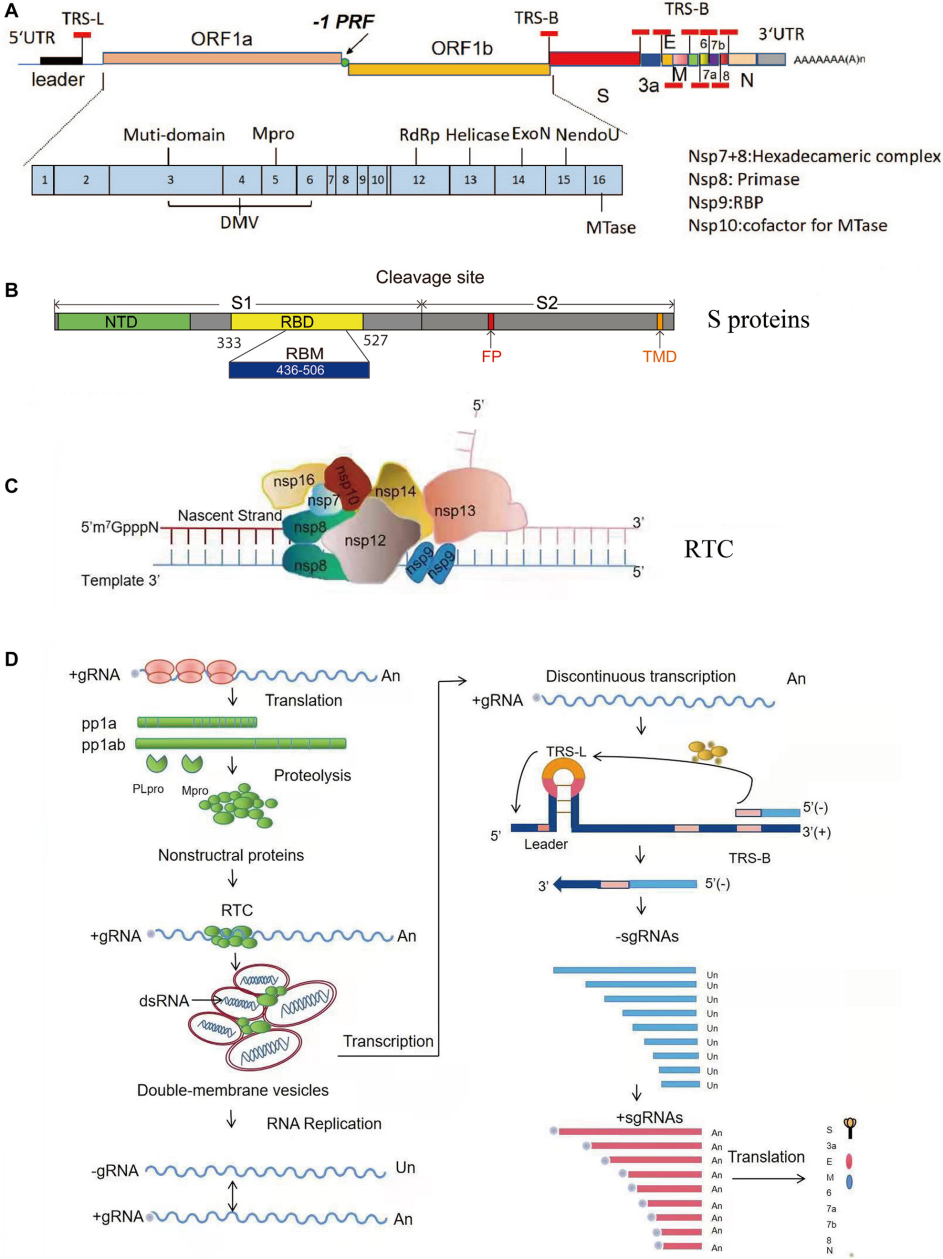
The scheme of genome organization and replication/transcription of SARS-CoV-2. A. The genome organization of SARS-CoV-2. The schematic diagram of the complete SARS-COV-2 genome is shown at the top. ORF1a and ORF1b encode polyproteins pp1a and pp1ab, respectively, which are further processed into 10 and 16 nsps by viral proteases. The expression of ORF1b is regulated by a ribosomal frameshifting mechanism. **–1PRF**: **–**1 programmed ribosome frameshifting element. The leader and body copies of transcription-regulating sequences (TRS-L and TRS-B, respectively) are indicated by short thick red lines. The functions of important nsps are indicated in the scheme. **UTR**, untranslated regions; **Mpro**, main protease; **RBP**, RNA binding protein; **RdRp**, RNA-dependent RNA polymerase; **S**, spike protein; **E**, envelop protein; **M**, membrane protein; **N**, nucleocapsid protein. B. **The schematic diagram of S protein of the SARS-CoV-2 S protein. NTD**, N-terminal domain; **RBD**, receptor-binding domain; **FP**, fusion peptide; **TMD**, transmembrane domain; **cleavage site**, furin cleavage site. C. A hypothetical model of the replicase and transcriptase complex of SARS-CoV-2. The diagram above shows how the replication-related proteins form an RTC. Nascent RNA is synthesized at the nsp12 RdRp domain. The nsp7 and nsp8 form the primase complex, nsp9 is a single-stranded binding protein and forms a dimer in the complex. Formation of the 5′ cap is catalyzed by nsp13, nsp14, nsp10, and nsp16. The locations of these proteins in the model are based on structural and functional analyses. D. A schematic of SARS-CoV-2 replication and transcription. Viral RNA replicates in the cytoplasm. ORF1a and ORF1ab are translated from the genomic RNA to produce pp1a and pp1ab polyproteins, which are then cleaved by viral papain-like protease (PLpro) and Mpro. Nsp 3, 4, and 6 are responsible for remodeling cellular membranes to form double-membrane vesicles (DMVs) where viral replication and transcription occur. The positive-sense genome is used as the template to produce full-length (−) RNA copies, which are used as templates for making full-length (+) RNA genomes. Negative-stranded sub-genomic RNAs (–sgRNAs) are synthesized through a unique discontinuous transcription mechanism in which fusion and transfer of a leader RNA sequence to body RNAs occur at transcription-regulating sequences (TRSs) with the help of the viral N protein and host proteins. The –sgRNAs serve as templates for sub-genomic RNAs (+sgRNAs) that are capped and polyA-tailed, and, despite many ORFs, only the closest ORF is typically translated.

SARS-CoV-2 is a single-stranded, positive-sense RNA virus. The genome of SARS-CoV-2 is approximately 29.9 kb with a cap structure at the 5′ end and a poly-A tail at the 3′ end [5], similar to host cellular mRNA; and it has 13–15 open reading frames (ORFs) flanked by 5′ and 3′ untranslated regions (UTRs) [6, 7], which contain cis-elements essential for RNA synthesis (Figure 1A). ORF1ab occupies two-thirds of the viral genome and is synthesized as a single polyprotein (Figure 1A) and then cleaved into 16 nonstructural proteins (nsps) by viral proteases encoded in nsp3 and nsp5. Most nsps are essential for the formation of the viral replication and transcription complex (RTC) [8]. The remaining one-third of the viral genome encodes four viral structural proteins: the spike protein (S), an envelope protein (E), membrane protein (M), and nucleoprotein (N), and several viral accessory proteins, including ORF3a, ORF3b, ORF6, ORF7a, ORF7b, ORF8, ORF9b, and ORF10 [6, 7]. The S protein is essential for binding its entry receptor, angiotensin-converting enzyme 2 (ACE2), and contains two subunits, S1 and S2, separated by a furin cleavage site (Figure 1B). S1 can be further divided into two domains, N-terminal domain (NTD) and receptor-binding domain (RBD). After binding to their receptor, S proteins may mediate membrane fusion either at the cell plasma membrane directly or at lysosomal membranes after internalization through endocytosis, depending on the availability of appropriate host proteases [9, 10]. The E and M proteins are required for effective virus assembly and budding, and the N protein binds to the viral genome and forms a helical ribonucleocapsid (RNP) that is essential for virus assembly [11]. Viral accessory proteins are not required for virus replication in cell culture, but they are suspected of playing important roles in viral pathogenesis in the natural host. Not all ORFs listed here have been experimentally verified, and the exact number of accessory proteins encoded in the SARS-CoV-2 genome remains to be determined [6, 7, 12, 13]. In addition, the SARS-CoV-2 genome might encode other unknown ORFs involved in the regulation of viral replication or host immune responses [6].

Once SARS-CoV-2 enters a cell, viral RNA replication and transcription, which are controlled by RTC, begin. Like many other positive-sense RNA viruses, SARS-CoV-2 RNA synthesis likely occurs inside the endoplasmic reticulum (ER)-derived double-membrane vesicles (DMVs) [14]. DMVs may not only protect viral RNA replication intermediates from host cytosolic innate immune sensors but also provide a place with adequate concentrations of substrates required for RNA synthesis. Viral nsp3, nsp4, and nsp6 have been implicated in the formation of DMVs [14]. In the RTC, nsp12 serves as an RNA-dependent RNA polymerase (RdRp), catalyzing viral RNA synthesis with the help of two viral cofactors, nsp7 and nsp8 [15]. The nsp8 protein is a primase, and nsp7, nsp8, and nsp12 together form the core of the RTC (Figure 1C). The nsp9 protein forms a dimer and regulates the replication process. Viral nsp13 [16] and nsp14 [17] also play important roles in regulating viral RNA synthesis during elongation: nsp13 is a viral helicase [18], and nsp14 provides 3′–5′ exonuclease activity with a proofreading function [19]. Nsp13 and nsp14 also contribute to the 5′ capping of viral RNAs [18, 20]. The coronavirus capping machinery includes nsp10, nsp13, nsp14, and nsp16. Nsp13 provides RNA 5′-triphosphatase activity [18], nsp14 has N7-methyltransferase activity [20], nsp16 is a 2′-O-methyltransferase [21], and nsp10 acts as a cofactor for nsp14 and nsp16 [22, 23].

During viral replication, the positive-sense viral genome is used as the template to synthesize full-length negative-sense genome copies (Figure 1D). In return, negative-sense genomes serve as the templates for the generation of progeny viral RNA genomes, which can be translated to produce more nsps and RTCs. Viral structural proteins and accessory proteins are generated from individual viral subgenomic RNAs (sgRNAs), which result from a unique discontinuous transcription process during negative-strand synthesis, a hallmark feature of CoV replication and transcription [8, 24]. There are transcription regulatory sequences (TRS) located upstream of most ORFs in the coronavirus genome [8]. The TRS adjacent to the leader sequence in the 5′ UTR of the viral genome is named “TRS-L”, whereas all other TRSs are called TRS-“body” or TRS-B. In the case of SARS-CoV-2, the TRS sequence is “ACGAAC” [5], [6], [7]. During negative-strand RNA synthesis, the RTC likely pauses on specific sequences containing TRS-B and reinitiates synthesis at TRS-L (Figure 1D). The nascent negative-sense sgRNAs are then used as templates to synthesize positive-sense sgRNAs for the expression of structural and accessory proteins. The discontinuous transcription process likely involves interactions between complementary TRSs of the nascent negative-strand RNA (negative-sense TRS-B) and the positive-strand genomic RNA (positive-sense TRS-L) [8]. The exact molecular mechanism underlying discontinuous transcription remains elusive.

## Comparative genomics of SARS-CoV-2 and related CoVs

### Identification of SARS-CoV-2-related CoVs

Great efforts have been undertaken worldwide to trace the origin of SARS-CoV-2, but it remains elusive when and where SARS-CoV-2 originated. The current consensus is that it is extremely unlikely that a lab leak was the source of the pandemic virus [25]. Instead, many studies have supported the view that SARS-CoV-2 had a zoonotic origin and evolved in nature [26], [27], [28], [29]. Because the place of virus origin is usually different from the place of the first recognized outbreak [30] and investigating the origin of a virus can take tremendous time and effort [31, 32], further studies are needed to better understand the origin of SARS-CoV-2 [33].

Despite the zoonotic signatures observed in the SARS-CoV-2 genome, it remains unclear how this virus was transmitted from animals to human populations [28]. Nevertheless, recent studies have identified various CoVs in bats closely related to SARS-CoV-2 (termed SC2r-CoVs), including RaTG13 from *Rhinolophus affinis* [3], BANAL-20-236 from *R. marshalli* [34], BANAL-20-52, BANAL-20-116, BANAL-20-247, and RmYN02 from *Rhinolophus malayanus* [34, 35], Rc-o319 from *Rhinolophus cornutus* [36], RshSTT182 and RshSTT200 from *Rhinolophus shameli* [37], RacCs203 from *Rhinolophus acuminatus* [38], and BANAL-20-103 and RpYN06 from *Rhinolophus pusillus* [34, 39]. Bats are common natural hosts for CoVs [40], [41], [42], [43], supporting that SARS-CoV-2 likely had a bat origin.

**Figure 2:**
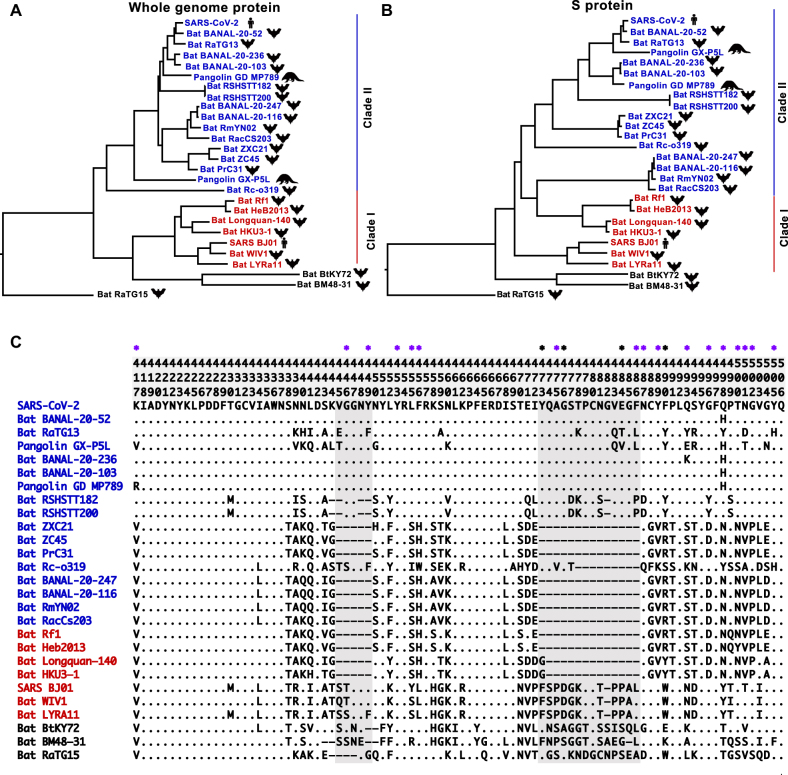
Phylogeny of SARS-CoV-2 and representative related CoVs. A. Phylogenetic tree reconstructed with concatenated protein sequences of nine conserved ORFs (orf1ab, S, ORF3a, E, M, ORF6, ORF7a, ORF7b, and N). B. Phylogenetic tree based on the S protein. In both **A** and **B**, the tree was reconstructed using MEGA X software [48], neighbor-joining method [49], and Jones-Taylor-Thornton (JTT) model [50]. The rate variation among sites was modeled with a gamma distribution (shape parameter=1), and the pairwise deletion option was used to remove ambiguous sites (SC1r-CoVs, red; SC2r-CoVs, blue). C. Protein sequence alignment of the receptor-binding motif (RBM). Site positions are shown at the top; the 17 ACE2-contacting residues identified by Wang et al. [51] and Lan et al. [52] are labeled with purple stars, and another four residues identified by Wang et al. [51] are labeled with black stars. A dot means the amino acid in that position is identical to that in SARS-CoV-2, and two deletions in the RBM are highlighted in gray.

As shown previously [38, 44], the currently known CoVs in the sarbecovirus lineage of the β-CoV genus can be categorized into two major clades, with one clade clustering with SARS-CoV-2 (termed SC2r-CoVs) and the other clade grouping with SARS-CoV (SC1r-CoVs) (Figure 2A). Notably, several bat CoVs, including RaTG13, BANAL-20-52, BANAL-20-103, and BANAL-20-236, share −96% nucleotide sequence identity with SARS-CoV-2 [3, 34]. Divergence of SARS-CoV-2 and RaTG13 was inferred to have occurred roughly 50 years ago [45, 46], although the exact divergence time depends on the substitution rate assumed in various analyses. Of note, a recent large-scale survey of CoV in bats in China failed to find any SC2r-CoV sequences, indicating that active circulation of SC2r-CoV might be limited in China [47], [48], [49], [50], [51], [52].

Besides bats, SC2r-CoVs have also been detected in Malayan pangolins (*Manis javanica*) [53], [54], [55], [56]. Pangolin-derived SC2r-CoVs (pangolin-CoVs) can be further classified into two sublineages, pangolin-CoV-GDC [53], [54], [55] and pangolin-CoV-GXC, which were found during anti-smuggling operations by Guangdong and Guangxi customs, respectively. While pangolin-CoV-GDC and SARS-CoV-2 share genomic sequence similarity of 92.4% [53], [54], [55], pangolin-CoV-GXC and SARS-CoV-2 show 85.5% nucleotide identity [56]. Both sublineages of pangolin-CoVs were isolated from *M. javanica*. However, a recent study identified an SC2r-CoV, MP20, from *M. pentadactyla* that likely originated from Southeast Asia [57]. MP20 is closely related to pangolin-CoV-GXC. An intra-host variant analysis revealed that the genetic diversity of pangolin-CoVs was substantially higher than expected, suggesting that pangolins might be the natural hosts of SC2r-CoVs [57]. The S proteins of viruses in both pangolin-CoV sublineages can bind hACE2 [44, 58], [59], [60], pointing to the potential risk of zoonotic transmission of pathogenic SC2r-CoVs from pangolins to humans.

### Roles of natural selection in SARS-CoV-2 and SC2r-CoVs divergence

The substitution rate at synonymous substitution sites in protein-coding regions (nucleotide changes that do not alter protein sequences) is routinely used as a proxy for the rate of neutral evolution. The synonymous substitution rate of SARS-CoV has been estimated to range from 1.67 to 4.67 × 10^−3^ substitutions/site/year [61], whereas the evolutionary rate of MERS-CoV was estimated at about 1.12 × 10^−3^ substitutions/site/year [62]. In comparison, the substitution rate in SARS-CoV-2 was roughly on the order of 10^−3^ substitutions/site/year [63], [64], [65], [66], [67], [68], although the exact rate has varied slightly across studies. These results indicate that SARS-CoV-2 has an overall evolutionary rate similar to SARS-CoV and MERS-CoV. Nevertheless, as observed in other CoVs [69], the evolutionary rate is heterogeneous across SARS-CoV-2 genes. For example, in comparing SARS-CoV-2 with SC2r-CoVs, we found considerable differences in synonymous substitution rates across genes, with the S gene showing a much higher evolutionary rate than other genes [70].

Comparative genomics has revealed how natural selection shaped the genome-wide differences between SARS-CoV-2 and SC2r-CoVs. Comparison between dN (nonsynonymous substitutions per nonsynonymous site) vs. dS (synonymous substitutions per synonymous site) values in coding regions provides a measure of the selective pressure on protein evolution, with a dN/dS (*ω*) value>1 indicating positive selection, *ω*=1 indicating neutral evolution, and *ω*<1 indicating purifying selection. By comparing the sequences of 13 protein-coding genes between SARS-CoV-2 and other closely related CoVs, we [70] found that in all pairwise comparisons, ω values ranged between 0.044 and 0.124, suggesting strong negative selection at nonsynonymous sites. Similar patterns have been observed in other studies [46, 71, 72]. Because nonsynonymous sites are under stronger negative selection than synonymous sites, analysis of sequence differences without separating these two classes of sites may underestimate the extent of molecular divergence several-fold. For instance, between SARS-CoV-2 and RaTG13, nucleotides genome-wide differ by −3.8%; however, the average dN is 0.78%, and dS is 16.8% (*ω*=0.0465), which means that, on average, 95.35% of nonsynonymous mutations that change protein sequences were removed by natural selection as SARS-CoV-2 and RaTG13 diverged. Although phylogenetic reconstruction using concatenated protein sequences indicates that some CoVs collected from bats in North Laos (BANAL-20-103 and BANAL-20-236) are more distantly related to SARS-CoV-2 than RaTG13 [34], the dS values from a comparison of SARS-CoV-2 and these two CoVs (0.1524, and 0.1577 for BANAL-20-103 and 236, respectively) tend to be slightly lower than those from a comparison of SARS-CoV-2 and RaTG13 (0.1682). Teasing apart the effect of natural selection can yield a better understanding of the phylogenetic relationships of CoVs (Table 1).

**Table 1:** The molecular divergence between SARS-CoV-2 and SC2r-CoVs.

	dN	dS	dN/dS
Bat RaTG13	0.0078	0.1682	0.0465
Bat BANAL-20-52	0.0059	0.1322	0.0447
Bat BANAL-20-103	0.013	0.1524	0.0855
Bat BANAL-20-116	0.0347	0.1967	0.1762
Bat BANAL-20-236	0.0133	0.1577	0.0841
Bat BANAL-20-247	0.0352	0.1988	0.177

Although the purifying selection is the predominant force governing the evolution of SARS-CoV-2 and SC2r-CoVs, signals of positive selection were also detected in nonsynonymous sites. By carrying out a CODEML analysis, we previously identified 10 nonsynonymous sites that showed strong signals of positive selection during the evolution of SARS-CoV-2 and other SC2r-CoVs [70]. Interestingly, five of these putative positively selected sites are located in the S protein (sites 46, 183, 439, 483, and 493), and three of them are located in the RBD of the S protein (439, 483 and 493). Using a similar analysis, Damas et al. identified three putative positively selected sites (455, 483, and 494) [73], and Cagliani et al. [72] found strong evidence of positive selection at seven sites, including six in the S protein (483, 484, 486, 490, 493, and 494). Sites 493 and 494 of the S protein were inferred to be positively selected in two of these studies, and site 483 was inferred to be positively selected in all three studies. Some of these inferences might have led to false positives, as the assumptions of CODEML were violated in analysis [74]. Therefore, functional studies are needed to investigate the consequences of these amino acid changes.

## Evolution of RBD and possible co-evolution with ACE2

### Deletions and possible recombination in the RBD

Compared with the phylogenetic tree based on protein alignments of all the conserved genes (Figure 2A), a considerably different tree was obtained when only S gene sequences were used for phylogenetic reconstruction, as some CoVs in the SC2r-CoV clade (e.g. RmYN02 and RAcCS203) grouped with viruses in the SC1r-CoV clade (e.g. Rf1 and HeB2013) (Figure 2B). This discrepancy might result from differences in genealogies of the S gene from those of other parts of the genome, as evidence of recombination is commonly observed in CoVs [5, 75, 76]. This pattern is manifested in the RBM (sites 436–506 of the S protein) of the RBD (Figure 2C).

There are two deletions in the RBMs of the RBD: deletions 1 (sites 445–449) and 2 (473–486). These deletions commonly coexist in coronavirus lineages such as RmYN02 and RacCs203. Previous analyses demonstrated that deletions in this sequence abolish the capacity of RBDs of RmYN02 and RacCs203 to bind to hACE2 [38, 44]. However, deletion 2 seems to be more important, because S proteins with deletion of sites 445–449 alone, such as that in RsYN04, retain some to bind to hACE2 [39]. The deletions are interspersed in SC1r-CoVs and SC2r-CoVs; intriguingly, however, viruses with both deletions also have highly similar sequences flanking these deletions (Figure 2C), suggesting that these deletions might have one single origin rather than multiple independent origins. Recombination may have shaped this discontinuity in the distribution of deletions within the phylogeny.

### Amino acid changes in RBDs of bat SC2r-CoVs

There are at least 17 amino acid residues in the RBD of the SARS-CoV-2 S protein that interact with hACE2 [51, 52] (Figure 2C). Eight (Y449, Y453, N487, Y489, G496, T500, G502, and Y505) of these 17 residues are conserved between S proteins of SARS-CoV and SARS-CoV-2, and 11 of the 17 residues (K417, G446, Y453, L455, F456, A475, N487, Y489, G496, T500, and G502) are identical between the S proteins of SARS-CoV-2 and RaTG13 (Figure 2C). Nevertheless, the S protein of RaTG13 can still use hACE2 as its entry receptor, although the entry efficiency is lower than that of SARS-CoV-2 [77], [78], [79]. These results highlight the plasticity of RBD/hACE2 interactions. Recently, CoVs isolated from bats in North Laos were found to show very high homologies with SARS-CoV-2 [34]. The S proteins of BANAL-20-52 and BANAL-20-236 share amino acid sequence identities of over 98.4 and 90.6%, respectively, with that of SARS-CoV-2. Among the 17 ACE2-contacting residues in the S protein, there is only H498 in BANAL-20-52 and K493 and H498 in BANAL-20-236 that differ from those residues in SARS-CoV-2, indicating that both S proteins likely use hACE2 as the entry receptor. Interestingly, although deletion 1 is found in S proteins of bat RSHSTT182 and RSHSTT200 CoVs found in Cambodia, several critical ACE2 contact residues are preserved, including Q493, Q498, N501, and Y505 [37]. While the S protein of RSHSTT200 CoVs failed to bind to hACE2, it could bind *R. shameli* bat ACE2 for virus entry [37]. Similar to bat RSHSTT182 and RSHSTT200 CoVs, the S protein of RaTG15, a coronavirus isolated from *R. affinis,* has a short deletion 1 (Figure 2C). However, there are 10 residues (sites 417, 449, 475, 486, 487, 493, 498, 500, 501, and 502) and one deletion (site 446) in the 17 ACE2-contacting sites that differ between SARS-CoV-2 and RaTG15. Experimental results reveal that these differences, possibly combined with the effect of deletion 1, might be responsible for the loss of binding affinity to hACE2 [44]. In summary, there are substantial differences in the critical functional sites in RBDs across bat CoVs, but only some changes may be associated with differences in the entry of human cells.

### Amino acid changes in RBDs of pangolin-CoVs

Neither of the two currently known pangolin-CoV sublineages has any deletions in the RBM (Figure 2C). Although the pangolin-CoVs are more distantly related to SARS-CoV-2 than RaTG13, previous studies have revealed almost identical amino acid sequences in the RBD region between pangolin-GDC-CoV and SARS-CoV-2 [55, 80]. As shown in Figure 2C, only two (417 and 498) out of the 17 ACE2-contacting residues differ between the RBDs of pangolin-GDC-CoV and SARS-CoV-2. It seems likely that the identical residues in SARS-CoV-2 and pangolin-GDC-CoV resulted from convergent evolution or recombination [56, 70, 80]. Notably, 12 of these 17 residues (G446, Y449, Y453, L455, F456, A475, N487, Y489, G496, T500, G502, and Y505) are identical between pangolin-GXC-CoV and SARS-CoV-2 (Lam et al. [56]). Despite differences in key functional residues between the two sublineages of pangolin-CoVs (Figure 2C), the RBDs of both pangolin-CoV sublineages bind efficiently to hACE2 [44, 81]. In addition, the RBDs of pangolin-CoVs seem to indicate a broader host range than those of SARS‐CoV‐2 [81]. Of note, S protein residue 498 differs across SARS-CoV-2 (Q), RaTG13 (Y), pangolin-CoV (H), BANAL-20-52 (H), and BANAL-20-236 (H), and introducing a Q498H substitution in the SARS‐CoV‐2 RBD expands its binding capacity to ACE2 of mice, rats, and European hedgehogs [28, 81].

### Evolution of ACE2 in animals and possible co-evolution with RBD

In addition to differences in the RBD region across SC2r-CoVs, sequence changes in the ACE2 receptor can influence RBD-ACE2 binding affinity. There are about 20 residues in ACE2 that interact with viral S proteins. Comparative genomic analyses revealed multiple amino acid changes in ACE2 across animals that putatively affect binding of the SARS-CoV-2 RBD to ACE2 [73, 82]. Bat *Rhinolophus macrotis* ACE2 (bACE2-Rm) exhibits a substantially lower affinity to the RBD of SARS-CoV-2 than hACE2 does [83]. A detailed analysis has revealed that residues 41 and 42 in bACE2-Rm play important roles in interactions of the receptor with SARS-CoV-2 RBD, with the Y41-Q42 combination yielding a high binding affinity and the H41-E42 combination resulting in a much weaker binding affinity [83]. While the S proteins of both SARS-CoV-2 and RaTG13 can bind to ACE2 of bat *R. affinis* (RaACE2), the binding affinity of SARS-CoV-2 RBD to RaACE2 is much weaker than that to hACE2 [28]. The RBD of RaTG15 shows clear discrepancies in binding to ACE2s from different species; it can bind to ACE2 of both *R. affinis* and Malayan pangolins, but fails to bind hACE2 [44]. More comprehensive studies are needed to dissect the complicated interactions between various RBDs of SC2r-CoVs and different ACE2 homologs, as well as to understand the mechanism of possible co-evolution between SC2r-CoVs and the animal host receptors.

## Mutational bias in the genomes of SARS-CoV-2 and SC2r-CoVs

Besides replication errors, several confounding factors such as host antiviral proteins and spontaneous chemical reactions can lead to mutations in the genomes of RNA viruses [69]. RNA editing enzymes, including adenosine deaminases acting on RNAs (ADARs) and apolipoprotein B mRNA editing enzyme, catalytic polypeptides (APOBECs), play important roles in the innate immune restriction to counter SARS-CoV-2 infection by inducing point mutations in SARS-CoV-2 genomes [6, 84, 85].

**Figure 3:**
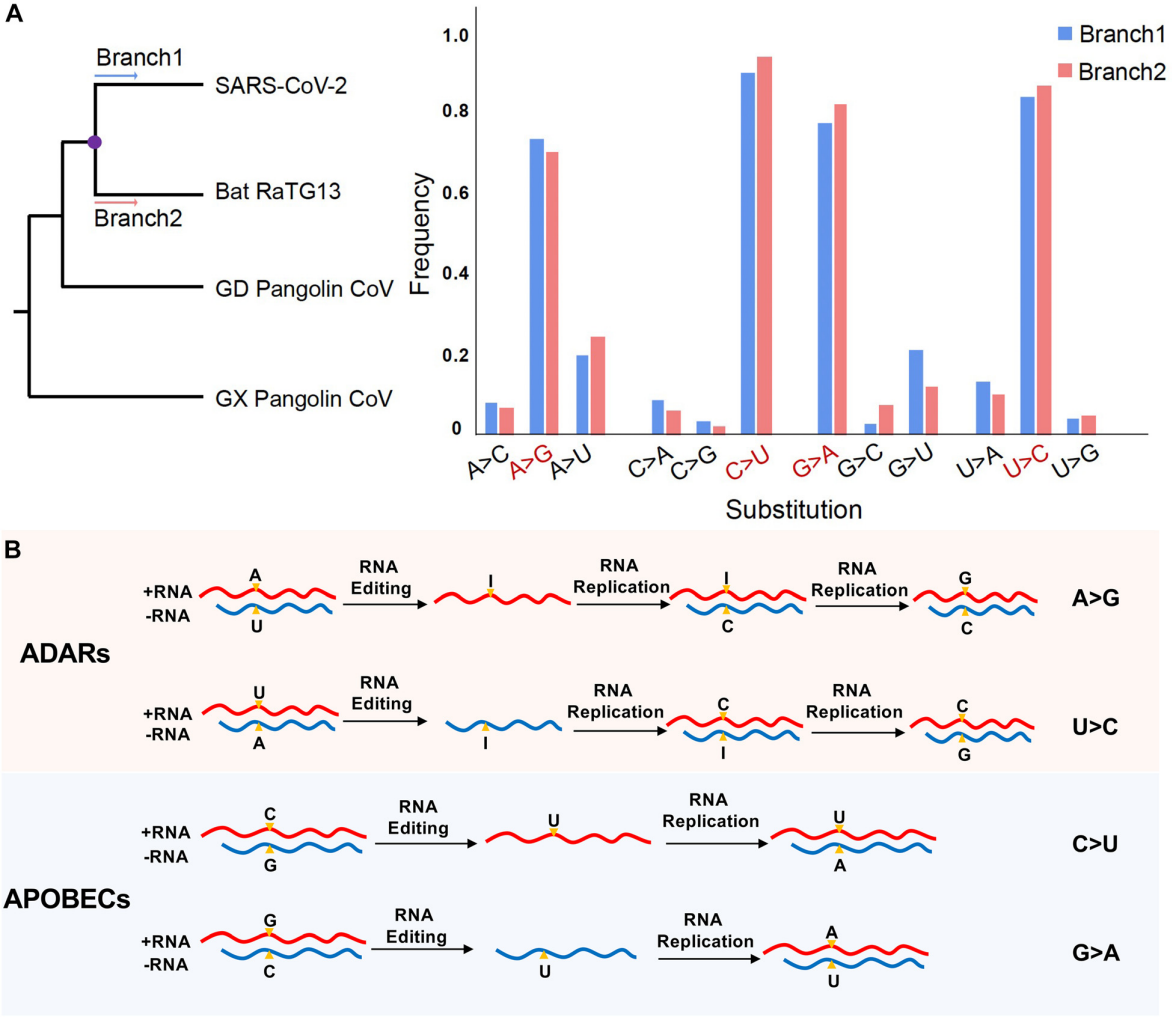
Model of possible RNA editing-induced mutational bias in SARS-CoV-2 evolution. A. Nucleotide substitution frequencies at synonymous sites in branches from the most recent common ancestor of SARS-CoV-2 and RaTG13 (the purple point in the phylogenetic tree on the left) to SARS-CoV-2 (B1) and RaTG13 (B2). B. A-to-I editing events catalyzed by ADARs in the sense or antisense strand of SARS-CoV-2 cause A-to-G or U-to-C mutations (upper panel); C-to-U editing catalyzed by APOBECs in the sense or antisense strand of SARS-CoV-2 cause C-to-U or G-to-A mutations (lower panel).

Several studies have shown excessive A-to-G and C-to-U mutations occurred during SARS-CoV-2 evolution [86], [87], [88], [89], [90], [91]. For example, one study examined the nucleotide substitution matrix from the most recent common ancestor of SARS-CoV-2 and RaTG13 to that of SARS-CoV-2 and RaTG13, and observed a strong nucleotide substitution bias at synonymous sites [14]. Specifically, A-to-G, C-to-U, U-to-C, and G-to-A substitutions were the most abundant when the ancestral nucleotide was A, C, U, and G, respectively (Figure 3A). It was proposed that the overabundance of C-to-U transitions in the SARS-CoV-2 genomes can be caused by the activity of APOBEC cytosine deaminases [92]. Here, we propose a model of possible RNA editing-induced mutational bias in SARS-CoV-2 evolution. Under this model, A-to-I editing events catalyzed by ADARs in the sense or antisense strand of SARS-CoV-2 cause A-to-G or U-to-C mutations; and C-to-U editing catalyzed by APOBECs in the sense or antisense strand of SARS-CoV-2 cause C-to-U or G-to-A mutations (Figure 3B). However, other mechanisms that might lead to similar observations could not be excluded.

## SARS-CoV-2 lineage analysis and the continuing evolution

Although SARS-CoV-2 has a proofreading mechanism, mutations remain inevitable during the replication of RNA viruses. By carrying out experimental evolution experiments with two circulating SARS-CoV-2 strains, a recent study estimates a genomic mutation rate of 2.9–3.7 × 10^−6^ mutation/site/cycle for SARS-CoV-2 under cell culture condition [93], which yields roughly 0.1 mutations/genome/cycle. With millions of SARS-CoV-2 genome sequences deposited in databases, including the Global Initiative on Sharing All Influenza Data (GISAID; https://www.epicov.org) [94, 95] and National Genomic Data Center of China (https://ngdc.cncb.ac.cn/) databases, hundreds to thousands of single nucleotide variants (SNVs) have been identified [70, 96], [97], [98], [99], [100]. The roles of individual SNVs in zoonotic origin, evolution, and transmission of SARS-CoV-2 have become the focus of many studies [25, 64, 101–104]. Based on 103 available SARS-CoV-2 genomes, we found that SARS-CoV-2 could be divided into two major lineages, L and S, early in the COVID-19 pandemic [70]. The distinction between L and S lineages depends on two SNV pairs at sites 8,782 and 28,144 with nearly complete linkage: C8782/U28144 for L and U8782/C28144 for S, with the reference genome (NC_045512) belonging to the L lineage. Residue 8,782 is encoded in the nsp4 gene. The C-to-U change at position 8,782 has no effect on the resulting amino acid, whereas residue 28,144 is encoded in the accessory protein ORF8 and the U-to-C substitution at position 28,144 leads to a codon switch from leucine (L) to serine (S). The “L” and “S” lineages are named because of leucine and serine residues, respectively, at position 28,144. Of note, the S lineage is considered to be ancestral to the L lineage when a tree is rooted by bat and pangolin CoVs as the outgroup [99]. Using Forster’s nomenclature, SARS-CoV-2 variants are classified into three lineages: A, B, and C. “A” lineage is equivalent to our “S” lineage, “L” lineage is further divided into “B” and “C” lineages. Moreover, based on these two sites and other SNVs, GISAID (http://gisaid.org) divides SARS-CoV-2 genomes into four major groups (S, L, V, and G), In contrast, Nextstrain (https://nextstrain.org) [105] categorized SARS-CoV-2 variants into five major clades (19A, 19B, 20A, 20B, and 20C). Finally, in the popular Pango nomenclature of SARS-CoV-2 (https://cov-lineages.org/index.html), classification of “A” and “B” lineages is also based on SNVs at positions 8,782 and 28,144, with “A” equating to “S” and “B” equating to “L”. Despite the substantial expansion of the number of viral genomes analyzed, the distinction between SARS-CoV-2 L and S lineages remains robust. For instance, among the 127,119 high-quality SARS-CoV-2 genomes we previously analyzed, 120,958 (95.15%) belonged to the L lineage, 5,950 (4.68%) belonged to the S lineage, and only 211 (0.17%) could not be accurately assigned to either the L or S lineage [106].

**Figure 4:**
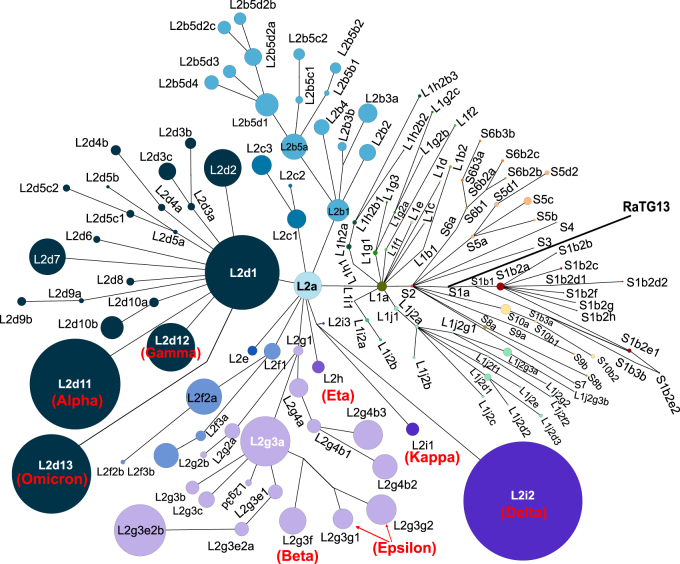
Updated haplotype network of SARS-CoV-2 sublineages based on the L/S nomenclature system [106]. Except for L2d11, L2i2, and L2d13 the size of each dot indicating a sublineage was scaled to the number of genomes in that sublineage (Sizes of L2d11, L2i2, and L2d13 have been reduced for better visualization). Some representative variants of concern (VOCs) and variants of interest (VOIs) are labeled in red in the network (Alpha: L2d11; Beta: L2g3f; Gamma: L2d12; Delta: L2i2; Omicron: L2d13; Epsilon: L2g3g1 + L2g3g2; Eta: L2h; Kappa: L2i1).

Given the rapid accumulation of publicly available SARS-CoV-2 genome sequences, analyzing the relatedness of SARS-CoV-2 genomes using traditional phylogenetic methods is a significant challenge. The power of a phylogenetic analysis can also be limited for tracing genealogies when ancestral, and descendent sequences are pooled [107, 108]. Further, because viruses often evolve through multifurcation, especially when superspreaders play a role in transmission [109], the hierarchical bifurcating assumption in the traditional phylogenetic inference may be violated. As an alternative, we have proposed determination of the lineage of a SARS-CoV-2 genome combined with haplotype network analysis to trace geneologies [106]. Specifically, based on L/S delineation according to variants at sites 8,782 and 28,144, we further divided the L lineage into two major sublineages (L1 and L2) using three tightly linked variants at sites 3,037, 14,408, and 23,403, and further categorized SARS-CoV-2 strains into 130 sublineages with SNVs at 201 additional sites. Our lineage designation system is hierarchical and can be easily expanded with new variants that might arise and become prevalent. In Figure 4, we incorporated the characteristic mutations in representative variants of concern (VOCs) and variants of interest (VOIs) and updated the haplotype network of SARS-CoV-2 sublineages based on the previous L/S nomenclature system [106].

## Important SARS-CoV-2 variants and their biological, immunological, and clinical characteristics

### Important variants of concern (VOCs) and variants of interest (VOIs) and their biological, immunological, and transmissibility

**Figure 5:**
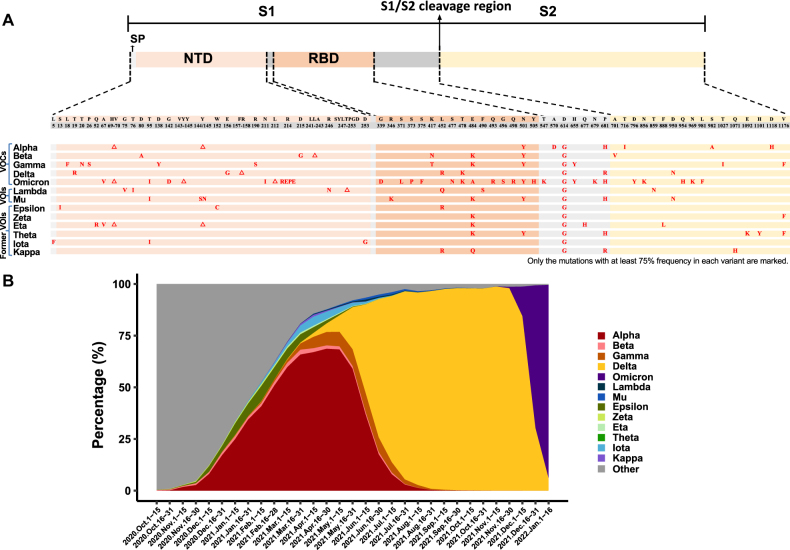
Prevalence and characteristic mutations in the S protein of VOCs and VOIs. A. Characteristic mutations (relative to the reference genome NC_045512) in the S proteins of VOCs and VOIs. In each variant lineage, the mutations that have a frequency of ≥75% in the sequences of that lineage are shown in red (Data were taken from Outbreak.info, last accessed on 20 January 2022). △, deletion; A, alanine; R, arginine; N, asparagine; D, aspartic acid; C, cysteine; E, glutamic acid; Q, glutamine; G, glycine; H, histidine; I, isoleucine; L, leucine; K, lysine; F, phenyl-alanine; P, proline; S, serine; T, threonine; Y, tyrosine; V, valine. Note that a 3-amino-acid insertion (EPE) occurred after R214 of the Omicron Spike protein. B. Prevalence of VOCs and VOIs over time. SARS-CoV-2 genomes with collection date information in the GISAID database (6,977,884 in total, deposited between October 1st, 2020 and January 16th, 2022) were used in the analysis. The number of genomes was updated at two-week intervals.

Amino acid changes in the S protein affect virus infectivity and host immune responses against the virus [110]. For instance, an N234Q change promotes resistance to neutralizing antibodies, whereas an N165Q change makes the virus more sensitive. A D614G mutation in the S protein (A23403G) and C3037U and C14408U greatly enhances virus infectivity and transmission. Based on these mutations, L1 and L2 sublineages can be defined [106]. The L1 sublineage carries the ancestral D614 variant, whereas the L2 sublineage has the G614 mutation. As shown in Figure 5B, SARS-CoV-2 sublineages exhibit substantial differences in temporal distributions; currently, the L2 sublineage is dominant worldwide.

Population genetic analyses have revealed that the D614G change is driven by positive selection [111–113]. Precisely, selective coefficients of G614 over D614 were estimated to be 0.31–0.55 [112] and 0.06–0.56 [113] when considering SARS-CoV-2 strains sampled worldwide. Strains found in the initial outbreak mainly carry D614, whereas G614 strains became dominant during the pandemic [106, 111, 112]. Although residue 614 itself is not located on the surface of the RBM, the G614 S protein seems to adopt a more open conformation that allows the S protein to bind to hACE2 more efficiently [114]. Numerous experiments have shown D614G enhancement of viral replication in human lung epithelial cells and primary human airway tissues, leading to high virus titers in the upper respiratory tract and greater transmissability, that result from the greater infectivity and virion stability conferred by this change [115–121]. However, the open conformation of the S protein may also increase susceptibility to antibody neutralization [122]. Similar to D614G, some emerging variants alter receptor binding affinity, reduce antibody neutralization activity, and affect the T cell response, potentially impacting COVID-19 diagnosis, treatment, and vaccine effectiveness globally.

To mitigate the potential impacts of some important variants and communicate differences between variants more easily to the public, WHO developed a classification system that defines two classes of SARS-CoV-2 variants, VOIs and VOCs. A VOI is defined as a SARS-CoV-2 variant with specific genetic markers that are known or predicted to be associated with an increase in virus transmissibility, disease severity, or immune escape, or a decrease in the efficacy of treatments and diagnostic assays. A VOC is defined as a variant that shows evidence of an increase in transmissibility, disease severity, or immune escape. Both VOIs and VOCs are named with Greek alphabet letters, and the list is periodically adjusted as the pandemic progresses. As of January 18th, 2022, there are five VOCs, Alpha, Beta, Gamma, Delta and Omicron; two VOIs, Lambda and Mu; and six formerly circulating VOIs, Epsilon, Zeta, Eta, Theta, Iota, and Kappa. Figure 5A shows characteristic mutations in the S protein that define VOCs. In Figure 5B, we present the bi-weekly worldwide prevalence of these variants. In the following, we will briefly summarize the current understanding of the five VOC lineages, with a focus on the S protein.

The Alpha (B.1.1.7) variant has two deletions (sites 69–70 and site 144) and seven amino acid changes (N501Y, A570D, D614G, P681H, T716I, S982A, and D1118H) in the S protein. This lineage shows greater transmissibility than the SARS-CoV-2 variant circulating prior to its appearance [113, 123]. The Alpha lineage showed a modest increase in resistance to neutralizing antibodies, but the E484K substitution in a small fraction of strains in the Alpha lineage (∼0.3%) was found to facilitate immune escape [124–127].

The Beta (B.1.351) variant carries three mutations in the RBD (K417N, E484K, and N501Y), three in the N-terminal domain (D80A, D215G, and a deletion of sites 241–243), and one mutation in the S2 subunit (A701V). This variant was first identified in South Africa in October 2020, and it spreads rapidly in Africa due to a selective advantage putatively resulting from enhanced transmissibility [128] or immune escape [125, 129, 130].

The Gamma (P.1) variant has three mutations (K417T, E484K, and N501Y) in the RBD and nine other mutations (L18F, T20N, P26S, D138Y, R190S, D614G, H655Y, T1027I, and V1176F) in the S protein. The three mutations in the RBD confer an increased binding affinity to hACE2 to strains in this lineage; strains in the gamma lineage may be 1.7- to 2.4-fold more transmissible than previously circulating non-Gamma strains [131]. In addition, virus strains in the gamma lineage have shown increased resistance to neutralizing antibodies [132, 133].

The Delta (B.1.617.2) lineage has one deletion (sites 157–158) and seven amino acid mutations in the S protein (T19R, E156G, L452R, T478K, D614G, P681R, and D950N). The infection rate with Delta strains is significantly higher than strains from other lineages. Delta is currently the most prevalent variant circulating worldwide. This variant is considerably less sensitive to serum neutralizing antibodies than pre-existing strains that only bear the D614G substitution [134]. The Delta lineage shows more efficient replication in airway organoid and human airway epithelial cells and spike-mediated entry than strains in the Alpha lineage [134]. The S protein of the Delta variant appears to mediate faster membrane fusion than other variants [135], resulting in a higher virus load and faster transmission rate [136]. The L452R mutation in the RBM confers increased infectivity and neutralizing antibody resistance to this variant [137–144]. Interestingly, the L452Q mutation in the Lambda variant might have similar effects as the L452R change in the Delta lineage [145–147]. Additionally, the P681R mutation in the Delta lineage may enhance furin cleavage of the spike protein into S1 and S2 subunits, facilitating more efficient cleavage by TMPRSS2 and increased virus infectivity [148].

The Omicron variant (B.1.1.529) was first detected in patients traveling from South Africa in November 2021, and has rapidly expanded globally. A substantial number of changes have occurred in the S protein of Omicron variant, including three deletions (sites 69–70, sites 143–145, and site 212), one insertion (insertion of EPE after site 214), and 26 point mutations (12 of them are located in the RBD: G339D, S371L, S373P, S375F, S477N, T478K, E484A, Q493R, G496S, Q498R, N501Y, and Y505H). Despite the large number of mutations carried by the Omicron variant, it is not yet clear regarding the origin of this variant. It is possible that the Omicron variant might have evolved in human populations where the large-scale sequencing of SARS-CoV-2 genomes were not well carried out, or in immunocompromised people where many mutations in the SARS-CoV-2 genomes were allowed to accumulate, or resulted from cross-species transmission from animals that were infected with SARS-CoV-2 and accumulated adaptive mutations [149, 150]. Existing studies have shown that Omicron has a stronger immune evasion ability to neutralize antibodies than other mutant strains [151–154], but its pathogenicity might be significantly weakened [155–158].

In summary, the VOC lineages tend to be more infectious and have a greater capacity for immune escape. Although numerous mutations have been found across the SARS-CoV-2 genome, changes in the S protein have received the most attention. These residues determine not only receptor usage and host range but also serve as major targets for host immune responses and, therefore, vaccines. Of note, as the pandemic developed, many mutations have occurred in the genomes of the VOCs which differentiated each VOC lineage into many sublineages. In Figures S1–5, we presented the characteristic mutations in the S protein of the sublineages and their prevalence in each of the five VOC lineages. Although new S protein variants have emerged and disappeared throughout the pandemic and most changes have had little to no impact on critical characteristics of the virus, some mutations such as D614G, L452R, E484, and N501Y significantly affect virus infectivity and/or sensitivity to neutralizing antibodies. In particular, the N501Y mutation in the S protein is present in four of the five VOC variants (Alpha, Beta, Gamma, and Omicron). N501 interacts with several residues in hACE2 [79], and the N501Y mutation increases RBD binding affinity to hACE2 [159, 160]. Further studies are required regarding whether the N501Y mutations in different VOCs were descendants from one single mutation event or resultant from multiple independent parallel mutations. E484K is present in Beta and Gamma lineage strains and a small fraction (0.3%) of Alpha strains. The E484K mutation confers immune escape [161, 162], and a combination of E484K and N501Y can further increase resistance to antibody neutralization [161–165]. In addition, many other mutations in S protein, such as N234Q, N165Q, L452R, A475V, V483A, F490L, and combinations of these, may also affect neutralization by antibodies [144, 166, 167].

### Relationship between SARS-CoV-2 variants and pathogenicity

Despite the relatively well-understood relationships between a handful of variants and infectivity and immune escape of SARS-CoV-2 [110], how the variants affect pathogenicity and clinical manifestations of COVID-19 in patients is not yet well understood. Previously, among 271 patients (73 S- and 198 L-lineage patients) diagnosed with COVID-19 early during the COVID-19 outbreak in Wuhan, S-lineage patients exhibited significantly worse clinical outcomes than L-lineage patients, and this pattern held even after excluding other risk factors [168]. However, the underlying molecular mechanism, i.e. how changes at sites 8,782 and 28,144 affect the replication and transmission of SARS-CoV-2, is not yet clear. Although the D614G change in the S protein is associated with increased infectivity, D614 and G614 SARS-CoV-2 variants do not appear to differ significantly in pathogenicity or clinical severity in patients [113] or in their pathogenicity in hamsters [169].

Mixed results were obtained regarding whether the COVID-19 patients infected with the Alpha variant [170–173] or Beta variant [128, 174] exhibit more severe disease than those infected with the prior SARS-CoV-2 strains, despite these two VOCs tending to show increased infectivity. Viral infection experiments of animal models yielded mixed results as well. Both the Alpha and Beta variants were shown to be 100-fold more lethal than the original SARS-CoV-2 bearing 614D in K18-hACE2 transgenic mice [175]. Similarly, in the hACE2-bearing mice, infection with the Beta variant resulted in more severe clinical symptoms and more weight loss than infection with prototype strain IME-BJ05 [176]. In contrast, very similar virus replication kinetics and amounts of virus shedding were observed in rhesus macaques infected with Alpha, Beta, and the variant containing only the D614G mutation; however, the Beta variant was found to be slightly less pathogenic than the other two variants in this host [177]. The Delta variant is more pathogenic than the prototypic SARS-CoV-2 strain in hamsters, and the P681R mutation in the Delta variant might be associated with this enhanced pathogenicity [178]. However, whether the Delta variant will induce more severe disease remains unclear. Altogether, it is challenging to decipher the relationship between SARS-CoV-2 variants and pathogenicity in COVID-19 patients, as multiple confounding factors such as age, gender, and underlying medical conditions affect symptoms and clinical severity of COVID-19 [111, 179–181]. Animal models have provided important insights into the phenotypical changes and pathogenesis of SARS-CoV-2 variants. However, outcomes in animal models affected by changes in ACE2 gene and protein sequences may not be recapitulated in humans.

## Driving forces and possible trends in the evolution of SARS-CoV-2

### Epistatic interactions between SARS-CoV-2 variants

**Figure 6:**
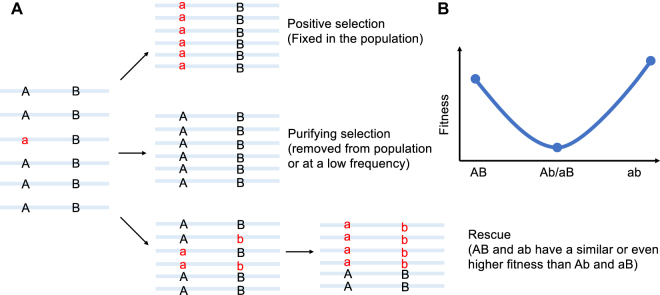
Schematic of epistatic interactions between two variants. A. A newly emerged variant (A>a) in the population might be harmful, neutral, or advantageous. Beneficial new variants are favored by natural selection and become fixed in the population very rapidly. In contrast, highly detrimental variants are removed by natural selection or persist in the population only at low frequencies. B. Under the epistatic interactions model, both A>a and B>b mutations are slightly deleterious. A virus with either an Ab or aB genotype has reduced fitness relative to the AB genotype. The virus with the ab genotype has a normal or even higher fitness. Thus, epistatic interactions can cause linkages between variants at the two sites to be maintained during viral evolution.

One salient observation is that many genetic variants of SARS-CoV-2 are tightly linked together. For instance, by analyzing SNVs in 121,618 high-quality SARS-CoV-2 genomes, we identified 202 pairs of mutations (133 sites) that exhibit strong linkage [106]. Intriguingly, despite the tremendous accumulation of SARS-CoV-2 genome sequences to date, variants at sites 8,782 and 28,144 continue to show an extremely high level of linkage, with C8782/U28144 variants specific to the L lineage and U8782/C28144 variants specific to the S lineage, and ∼0.2% of genomes unable to be accurately assigned to the L or S lineage. In addition, within the L lineage, variants at sites 3,037, 14,408, and 23,404 are tightly linked, with C3037/C14408/A23403 variants belonging to the L1 lineage, and U3037/U14408/G23403 variants belonging to the L2 lineage, and <0.2% of L genomes belonging to neither the L1 nor L2 sublineage [106]. These observations support the notion of extensive epistasis and advantageous compensatory mutations between tightly linked variants, as illustrated in Figure 6. Nevertheless, the molecular mechanisms underlying these epistatic interactions in viral transmission or pathogenicity are largely unknown. For instance, in mouse-adapted SARS-CoV-2 strains, double mutations at N501Y/Q493H conferred higher binding affinity for hACE2 than N501Y alone; however, triple mutations at N501Y/Q493H/K417N substantially decreased binding affinity for hACE2 [182], highlighting the importance of epistasis between these variants. Overall, the impacts of individual variants and combined effects of tightly linked variants on the transmission and pathogenicity of SARS-CoV-2 require further studies.

### Trends in SARS-CoV-2 evolution

One notable observation with SARS-CoV-2 is that one lineage tends to replace previously dominant lineages, with the Delta variant being predominant at present (e.g. Figure 5B). Nevertheless, the precise factors that shape this pattern are not clear. There are two competing theories on the evolution of virulence (i.e. pathogenicity) of a pathogen [183–186]. One long-standing view is that there is a trade-off between virulence and transmissibility, leading pathogens to evolve toward reduced virulence because weakening a host may reduce transmission. The alternative view is that a high level of virulence might be favored by natural selection if the more virulent strain compensates for the reduced transmission that can result from harming hosts. Continuous circulation of a SARS-CoV-2 variant is mainly driven by evolutionary forces that favor transmissibility and immune evasion rather than pathogenicity [177]. Further studies are needed to understand better the relationship between pathogenicity and infectivity caused by new SARS-CoV-2 variants that emerge.

### Cross-species transmission between humans and animals

An early study suggested that residue 372 of the SARS-CoV-2 S protein plays a vital role in virus adaptation in humans. An A372 (codon GCA at sites 22,676–22,678 of the genome) was found in nearly all 182,000 SARS-CoV-2 sequences surveyed [187], whereas T372 is found in the orthologous site (codon ACU or ACC) of all other SC2r-CoVs, indicating that the T372A change might have evolved during zoonotic transmission to humans. The T372A substitution not only abolishes N-linked glycosylation but increases affinity for hACE2, whereas A372 viruses replicate better than T372 viruses in human respiratory epithelial cells. Additionally, the T372A variant has shown evidence of positive selection upon examination of SARS-CoV-2 population data [187]. An N501Y substitution increases affinity of the S protein for hACE2, and viruses with Y at this site in the S protein show a broader host range, with mice susceptible to infection by N501Y strains [188]. In addition, mice can be infected with viruses carrying Q493K and Q498H changes in the S protein [189, 190].

Accumulating evidence indicates that SARS-CoV-2 can be transmitted from humans infected with COVID-19 to a wide range of mammals, including cats, minks, ferrets, lions, tigers, and white-tailed deer [191–193]. Notably, reverse zoonosis from humans to the animals can enable animals to become novel reservoirs for new SARS-CoV-2 variants that might be transmitted back to human populations with dramatic changes in infectivity or pathogenicity, as cross-species transmission can be accompanied by punctuated increases in mutations that may serve as raw materials for natural selection. For example, genome sequences prove that SARS-CoV-2 has been transmitted from humans to minks and then back to humans [194]. Thus, surveillance should be established to monitor possible back-and-forth transmission of SARS-CoV-2 between humans and animals.

## Concluding remarks and future perspectives

The COVID-19 pandemic has caused immense disruptions in the global economy and human health. Through comparative genomics, CoVs in animals that are closely related to SARS-CoV-2 has been identified. Evolutionary analysis of these CoV genomes has revealed strong signatures of positive and purifying selection as SARS-CoV-2, and SC2r-CoVs diverged. The interplay between SARS-CoV-2 and RNA editing mechanisms in hosts might have shaped the characteristic mutational bias in nucleotide changes during SARS-CoV-2 evolution. Genome sequencing has provided a powerful approach to identifying SARS-CoV-2 variants under putative positive selection, and defining lineages based on characteristic variants has facilitated studies of ongoing SARS-CoV-2 evolutionary dynamics. Although the molecular mechanisms and functional consequences of a few amino acid changes have been dissected, these changes have been mainly restricted to the S protein, with the causes and impacts of amino acid changes in other regions of SARS-CoV-2 genomes poorly understood. The epistatic relationship between amino acid changes and possible combined effects of such changes require further exploration. One observation of SARS-CoV-2 variants is that one lineage replaces previously dominant lineages; however, the factors underlying these patterns are not yet clear. It is also a challenge to decipher the relationship between pathogenicity and infectivity of variants. Further studies are needed to monitor the possible cross-species transmission of SARS-CoV-2 between humans and other animals as the pandemic develops.
